# Food as medicine: a quasi-randomized control trial of two healthy food interventions for chronic disease management among ambulatory patients at an urban academic center

**DOI:** 10.1017/S1463423623000579

**Published:** 2023-12-21

**Authors:** Peris W. Kibera, Nana A. Ofei-Tenkorang, Chanda Mullen, Aaron M. Lear, Elliot B. Davidson

**Affiliations:** 1 Center for Family Medicine, Cleveland Clinic Akron General, Akron, OH, USA; 2 Department of Pharmacy, Cleveland Clinic Foundation, Akron, OH, USA

**Keywords:** food as medicine, healthy eating, hypertension, medically tailored meals, nutrition counseling and education, obesity, prediabetes, produce programs, type 2 diabetes

## Abstract

**Background:**

Globally, poor nutrition is a driver of many chronic diseases and is responsible for more deaths than any other risk factor. Accordingly, there is growing interest in the direct provision of healthy foods to patients to tackle diet-linked chronic diseases and mortality.

**Aim:**

To assess the effect of two healthy food interventions in conjunction with nutrition counseling and education on select chronic disease markers, food insecurity, diet quality, depression, and on self-efficacy for healthy eating, healthy weight, and chronic disease management.

**Methods:**

This parallel-arm quasi-randomized control trial will be conducted between January 2022 and December 2023. Seventy adult patients recruited from a single academic medical center will be randomly assigned to receive either: i) daily ready-made frozen healthy meals or ii) a weekly produce box and recipes for 15 weeks. Participants will, additionally, take part in one individual nutrition therapy session and watch videos on healthy eating, weight loss, type 2 diabetes, and hypertension. Data on weight, height, glycated hemoglobin, blood pressure, and diabetes and blood pressure medications will be collected in-person at the baseline visit and at 16 weeks from baseline and via medical chart review at six months and 12 months from enrollment. The primary outcome of the study is weight loss at 16 weeks from baseline. Pre- and post-intervention survey data will be analyzed for changes in food insecurity, diet quality, depression, as well as self-efficacy for health eating, healthy weight, and chronic disease management. Through retrospective chart review, patients who received standard of care will be matched to intervention group participants as controls based on body mass index, type 2 diabetes, and/or hypertension.

**Findings:**

By elucidating the healthy food intervention with better health outcomes, this study aims to offer evidence that can guide providers in their recommendations for healthy eating options to patients.

## Introduction

Globally, poor nutrition is responsible for more deaths than any other risk factor, an outcome attributable to excess daily consumption of unhealthy foods and suboptimal consumption of nearly all healthy foods and nutrients (GBD 2017 Diet Collaborators, 2019). Consumption of a healthy diet, thus, remains the cornerstone for reducing diet-linked leading causes of mortality such as heart disease, hypertension, type 2 diabetes, and obesity (Joint WHO/FAO Expert Consultation, 2003; World Health Organization, 2013; Micha et al., 2017; GBD 2017 Diet Collaborators, 2019). A healthy diet consists of diverse macro- and micronutrients as well as water consumed in appropriate proportions to meet a person’s physiological needs. Such a diet consists of wholegrains, a variety of fresh or frozen (without additives) vegetables and fruits, nuts, herbs, beans and legumes, fish and lean animal protein such as chicken, and unsaturated fats, all with little-to-no added sodium or sugar (World Health Organization, 2018; Cena and Calder, 2020).

The cardiometabolic benefits of a healthy diet are welldocumented. Dietary fiber such as that found in vegetables, fruits, nuts, and wholegrains promotes satiety, has a positive effect on cholesterol levels and glycemic control, and is known to lower the risk of heart disease, stroke and death from all causes (Cena and Calder, 2020). Vegetables, fruits, nuts, and wholegrains also contain phytochemicals, which are important bioactive compounds that modulate cellular processes such as oxidative stress, nuclear transcription, fat metabolism, and inflammation (Liu, 2013). Phytochemicals have been associated with a reduced risk of chronic diseases (Vasmehjani *et al.*, 2021; Borgonovi *et al.*, 2022). Essential amino acids, which are important for maintaining a lean body mass, are derived from animal and plant protein sources. While some animal-based proteins contain saturated fats which might contribute to cardiometabolic diseases (Cena and Calder, 2020), animal-derived amino acids are generally considered to have health benefits (Jennings *et al.*, 2015; Lonnie *et al.*, 2018), with plant protein shown to confer greater and more pervasive protection against diet-linked chronic diseases (Luo *et al.*, 2014; Jennings *et al.*, 2015; Jennings *et al.*, 2015; Malik *et al.*, 2016; Shang *et al.*, 2017; Ahnen *et al.*, 2019). Unsaturated fats, including the polyunsaturated essential fatty acids, omega-3 and omega-6, promote normal growth and reproduction and are known to have cardiovascular, neurological, and anti-inflammatory benefits as well as to prevent or improve sarcopenia and insulin resistance (Luo *et al.*, 2014; Ding *et al.*, 2017; Manuelli *et al.*, 2017; Stella *et al.*, 2018; Zhang *et al.*, 2019). Seafood, particularly oily fish, nuts, and seeds are the main sources of omega-3 fatty acids.

The preponderance of evidence supporting the health benefits of proper nutrition has led to the espousal of a food is/as medicine paradigm in health care and beyond (Mozaffarian *et al.*, 2019; Tufts University Friedman School of Nutrition Science and Policy, 2023). In the United States, clinicians are increasingly at the forefront of food is/as medicine efforts (Veldheer *et al.*, 2020), and in recent years, such endeavors have taken the form of direct provision of healthy foods to patients. Providing healthy foods directly to patients offers many benefits, including imparting practical knowledge about nutritious food items, introducing or incentivizing healthy eating, and addressing barriers such as financial and neighborhood access to healthy foods (Downer *et al.*, 2020). Direct interventions include free or subsidized medically tailored meals, medically tailored groceries, and fresh produce referral or prescription programs. Medically tailored meals are fully prepared meals designed by a professional nutritionist following individualized assessment of the recipient. Medically tailored meals are often paired with nutrition counseling and are typically targeted toward medically and socially complex individuals such as those with cancer, HIV/AIDS or heart failure, or those unable to purchase or prepare meals (Downer *et al.*, 2020). Medically tailored groceries and produce prescription/referral programs, on the other hand, are similar in that they primarily target food-insecure individuals with, or are at risk for, diet-linked chronic conditions who are able to cook and prepare meals at home. In the former intervention, grocery items are selected by a nutritionist as part of the recipient’s treatment plan, while the latter intervention offers currency, for example, vouchers or debit cards, or connection to a vendor where produce can be redeemed for free or at a discount (Downer *et al.*, 2020).

## Background

In 2019, our hospital, a nonprofit urban academic center in the Midwest United States, conducted a Community Health Needs Assessment (CHNA) through which it identified the prevention and management of chronic diseases as a significant need in the communities it serves (Cleveland Clinic Akron General, 2019). In the tri-county area served by the hospital, cardiometabolic diseases were noted to be highly prevalent, and heart disease and hypertension were documented as leading causes of mortality. The CHNA, additionally, reported that the proportion of adults with obesity in its service area was above the national average. In Summit County, the hospital’s main catchment area, adult obesity rose from 25% to nearly 30% between 2015 and 2018, with poor nutrition cited as a driver of obesity and other chronic diseases. Barriers to healthy eating identified by community members were lack of time to make meals, lack of knowledge about preparing nutritious meals, and residing in a food desert (Cleveland Clinic Akron General, 2019).

Research has shown that direct provision of healthy foods to patients aids weight loss (Cavanagh *et al.*, 2017), improves glycated hemoglobin (hbA1c) (Berkowitz, Delahanty, *et al.*, 2019), alleviates food insecurity (Berkowitz, Delahanty, *et al.*, 2019), and reduces acute health care visits (Berkowitz *et al.*, 2018; Berkowitz, Terranova, *et al.*, 2019; Berkowitz, O’Neill, *et al.*, 2019). While past research has tested direct provision of healthy meals and fresh produce with different populations, to our knowledge, no study has compared these two interventions in a parallel-arm design (Nair, 2019) with research participants drawn from the same population. Our study seeks to compare these two interventions within the same patient population to help illuminate the more efficacious healthy food intervention in improving obesity, type 2 diabetes, and hypertension, compared to standard of care. Participants in the intervention arms will also take part in nutrition counseling and education through the nutrition center of a local university. The inclusion of nutrition counseling and education in our study aims to address information gaps, with the goal of improving participants’ self-efficacy for healthy eating and chronic disease management beyond the tenure of the study. The nutrition education component is based in social cognitive theory, which postulates that change in health behavior hinges upon increase in self-efficacy to perform the desired behavior (Bandura, 2004).

Our study will collaborate with two local organizations which will supply healthy foods to participants. The first organization, SimplyEZ, is a 25-year-old meal delivery company that provides healthy ready-to-heat-and serve meals, including low-sodium and diabetic-friendly options, designed by a dietitian to customers in the Midwestern United States. The second organization, Perfectly Imperfect Produce, has the primary mission of reducing food waste and improving healthy food access for all. The organization rescues and sells aesthetically imperfect but perfectly fresh produce rejected by grocers and, additionally, operates a website featuring recipes, produce preparation, and produce storage tips accessible to the public at no cost. Our study seeks to compare these two healthy food options – ready-made healthy meals and a produce box – within the same patient population to help illuminate the more efficacious healthy food intervention in improving obesity, type 2 diabetes, and hypertension, compared to standard of care.

## Methodology

### Study aims

1. The primary aim of this study is to compare absolute weight change among participants receiving daily healthy meals or produce box versus matched controls (standard of care) at 16 weeks from baseline:
  - This aim tests the hypothesis that receiving daily healthy meals will result in greater absolute weight loss compared to receiving a produce box or standard of care at 16 weeks from baseline.
  - This aim tests the hypothesis that receiving either daily healthy meals or a produce box will result in greater absolute weight loss compared to standard of care at 16 weeks from baseline.
2. The secondary aim of this study is exploratory and will compare daily healthy meals or produce box versus matched controls in improving chronic disease markers:
  - This aim tests the hypothesis that receiving daily healthy meals will result in greater improvement in hbA1c and blood pressure compared to receiving a produce box or standard of care box at 16 weeks, six months, and 12 months from baseline.
  - This aim tests the hypothesis that receiving either daily healthy meals or a produce box will result in greater improvement in weight, hbA1c and blood pressure compared to standard of care at 16 weeks, six months, and 12 months from baseline.
3. To assess whether receiving daily healthy meals or a produce box in conjunction with nutrition counseling and education results in improvement in food insecurity, diet quality, depression, self-efficacy for healthy eating and healthy weight, and self-efficacy for chronic disease management from baseline to 16 weeks.

### Study design

This study is a parallel-arm quasi-randomized control trial with nonequivalent matched controls, which will be conducted between January 2022 and December 2023. Seventy adult patients recruited from our ambulatory practice will be randomly assigned to receive either: i) daily healthy frozen meals or ii) a weekly produce box and recipes for 15 weeks. Each study arm will comprise 35 patients with a documented diagnosis of obesity (body mass index [BMI] equal to or greater than 30kg/m^2^) and one or more of the following conditions: pre-type 2 diabetes/type 2 diabetes or hypertension. Participants will complete an in-person baseline visit and a follow-up visit at 16 weeks from baseline, and their medical charts will be reviewed for trends in these biomarkers at six months and 12 months from baseline. Healthy meals or a produce box will be shipped to the home address of participants once a week. Healthy meals provided in the study are low-sodium and diabetic-friendly ready-to-heat and serve breakfast, lunch, and dinner items. The produce box provided in the study will comprise fresh vegetables and fruits. For participants receiving a produce box, recipes utilizing items contained in the box will be available at the vendor’s website.

All participants will complete one individual medical nutrition therapy session via phone with a dietitian. The individual medical nutrition therapy appointment will occur within two weeks of enrollment in the study. A total of four nutrition and health videos, each under 10 minutes in length, will be available for participants to view between weeks 3 and 15 of the study. The complete study procedures are outlined in Table 1. Participants will be required to view the videos on healthy eating and weight loss and, as applicable to their health status, videos on type 2 diabetes and hypertension (see Table 2). The Healthie application (https://www.gethealthie.com/?afmc=a46i) will be utilized for nutrition education programming. Through the Healthie application, participants will receive automated text and/or email reminders to view educational videos. This feature is designed to drive compliance and completion of the nutrition education component. A report will be generated to provide completion percentage and other details based on participant engagement. The Healthie application is compliant with the US Health Insurance Portability and Accountability Act.

**Table 1.** Schedule of study activities

Study activity	Baseline visit (W0)	Week 2 (W0–W2)	Week 3 – 15 (W3–W15)	Week 16 visit (W16 ± 7 days)	6 months	12 months
Informed consent process	x					
Anthropometric data (weight, height, and blood pressure)	x			x	x	x
HbA1c	x			x	x	x
Completion of study questionnaires	x			x		
Individual nutrition counseling session		x				
Nutrition education videos			x			

**Table 2.** Expected viewership of nutrition education videos

	Video title			
	Introduction to nutrition	Nutrition and weight loss	Nutrition and diabetes	Nutrition and hypertension
All participants	x	x		
Participants with pre-/type 2 diabetes			x	
Participants with hypertension				x
Participants with pre-/type 2 diabetes AND hypertension			x	

**Figure 1.**
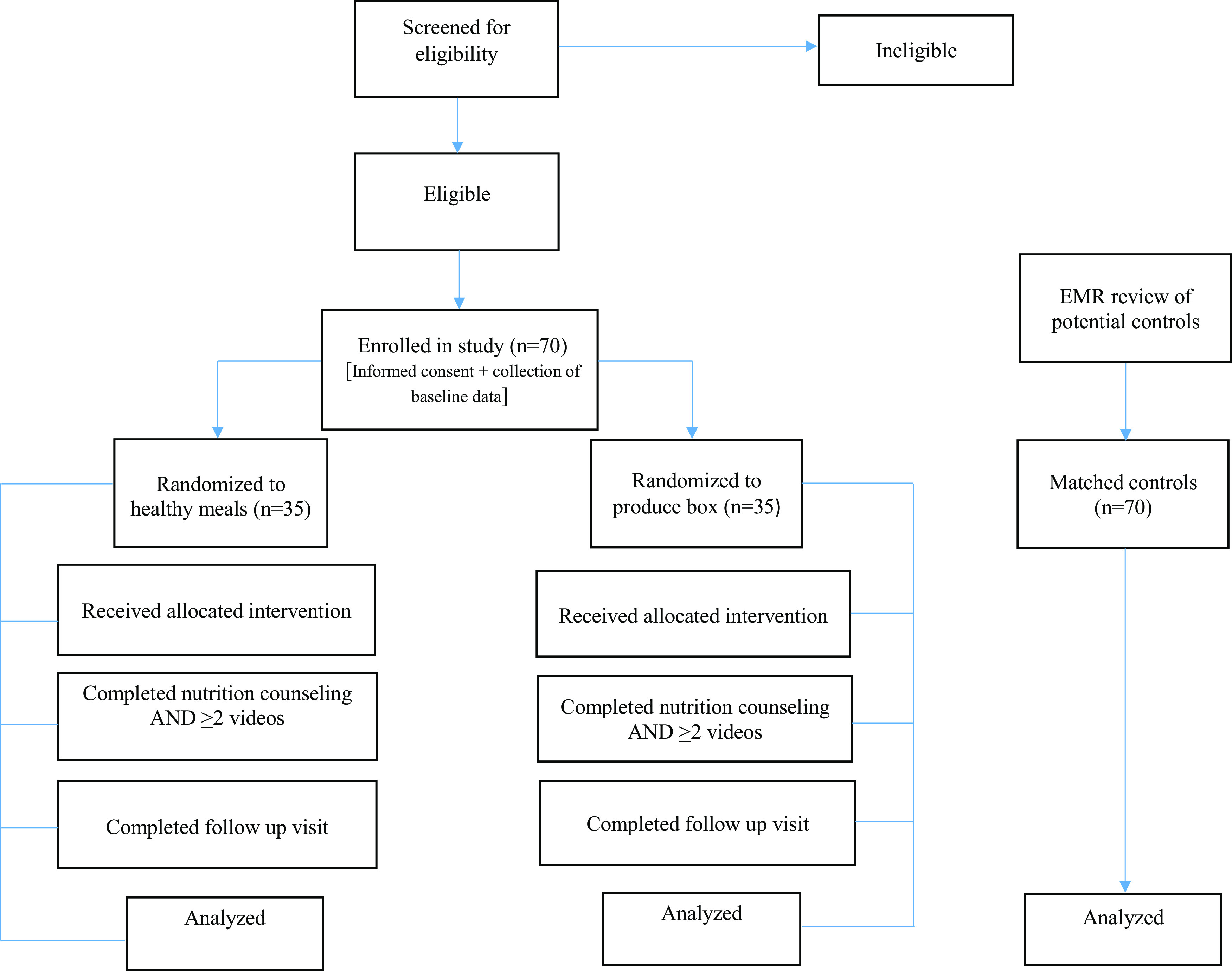
Flow chart of participant recruitment and completion of study activities.

The control group will comprise 70 participants matched to intervention group participants based on BMI, diagnosis of pre-type 2 diabetes/type 2 diabetes, and/or hypertension. Control group participants will be identified from the electronic medical record, Epic^®^, after enrollment of all intervention arm participants has been completed. A flow chart of participant recruitment and completion of study activities is shown in Figure 1. Our study protocol adheres to the SPIRIT guidelines for clinical trial protocols (Chan *et al.*, 2013) and is registered on ClinicalTrials.gov with identifier NCT05174078.

### Study population

Current patients at our ambulatory practice who are 18 years and older, and are of any gender and race, are eligible for enrollment in the study. Patients who have food allergies, are pregnant or planning to become pregnant, have no access to internet, or require English language interpretation will be excluded from participation. Additional exclusion criteria are participation in nutrition counseling with our partnering organization within the past six months, current participation in a structured weight loss program, or current use of weight loss medications. Medications prescribed for other conditions but that may have a weight loss effect, for example, metformin or GLP-1 agonists prescribed for diabetes treatment or topiramate prescribed for migraine headaches, will not result in the exclusion of potential participants.

### Recruitment strategy

A flyer inviting patients to participate in the study will be available in patient rooms as well as in patient waiting areas at our ambulatory practice. Flyers will have contact information (phone number and email address) for the study coordinator. Potential study participants will be informed about the study by a nurse or provider during their routine office health visits. If interested, potential participants may choose to meet with the study coordinator after their visit or they may call or email the study coordinator at the phone number provided on the tear-off of the study flyer. During phone calls or email correspondence with the study coordinator, eligible participants will receive detailed information about the study, and they will be invited to come to the practice to complete the consent process and baseline visit.

### Sample size determination

Similar past research conducted in the US (Berkowitz, Delahanty, *et al.*, 2019) was used to estimate a baseline weight of 92 ± 16 kg for the study population. In order to estimate the effect size of weight loss potential in the intervention groups, the CDC healthy eating standard range of 1 to 2 pounds weight loss per week was used. The estimate for weight loss in the healthy meals group was 2 pounds per week over 15 weeks, and the estimate weight loss for the produce group was 1 pound per week for 15 weeks. It was assumed that there would be no weight change in the matched controls (standard of care) group. A one-way AONVA sample size calculation was performed using the following parameters: mean weight (kg) [matched controls 92, healthy meals 78.4, Produce 85.2], standard deviation 16 for all groups, 0.80 power, and 0.05 alpha. This yielded a sample size of 28 per group. We assume a 10% attrition rate, bringing the sample size to 35 per group.

### Randomization

Randomization of participants will be conducted using the central randomization module available in the Research Electronic Data Capture (REDCap) program, a secure online database that will be utilized for data collection and management (Harris *et al.*, 2009, 2019). Randomization and allocation will occur in blocks of four. The allocation sequence was generated for a total of 80 participants using randomization software in the Sealed Envelope website (https://www.sealedenvelope.com/simple-randomiser/v1/lists) and uploaded into our study’s REDCap database. Following consent into the study and collection of all baseline data, study participants will be randomly allocated to the healthy meals or produce box group in a 1:1 ratio using the randomization sequence pre-entered into REDCap.

## Informed consent

This study was reviewed and approved by our hospital’s Institutional Review Board. The informed consent process will be conducted as the initial task during the baseline visit. Participants will be requested to offer a summary of their understanding of the study and study procedures prior to giving consent to participate in the study. Participants will also be given an opportunity to ask any questions, with their signature on the consent form solicited only after all questions have been answered to their satisfaction. Participants will receive the original signed consent form, and a copy will be stored by the project.

## Data collection

### Demographic and clinical data

Participants’ date of birth, sex, race, and ethnicity will be collected as part of demographic data. Objective health data that will be collected are weight, height, hbA1c, blood pressure, prediabetes/diabetes diagnosis and classification, hypertension diagnosis and classification, diabetes and blood pressure medications, and comorbid chronic conditions. These data will be collected in-person at the baseline visit and at 16 weeks from baseline and via chart review of routine care at six and 12 months from enrollment in the study.

### Survey data

Pre- and post-intervention surveys on food insecurity, diet quality, depression, as well as self-efficacy for healthy eating, healthy weight, and chronic disease management will be administered at baseline and at the 16-week follow-up visit. Surveys were compiled from the below validated instruments:

- The Hunger Vital Sign: assesses household food insecurity (*Hager et al.*, 2010).
- Eating at Americas Table Survey: assesses diet quality (*Borgonovi et al.*, 2022).
- Patient Health Survey Depression Scale: valid diagnostic and severity measure for depressive disorders (Kroenke *et al.*, 2009).
- Healthy Eating and Weight Self-Efficacy Scale: assesses individuals’ ability to maintain a healthy weight and eating pattern (Wilson-Barlow *et al.*, 2014).
- Self-Efficacy for Managing Chronic Diseases Scale: a six-item questionnaire that determines self-efficacy as one takes an approach to improve health behaviors to managing varying chronic diseases (Ritter and Lorig, 2014).

In addition, the post-intervention survey contains 11 non-validated items assessing adequacy, practicality, and satisfaction with healthy meals or produce box received by participants. These additional non-validated items were developed by the first author.

### Data analysis

The independent variables in this study are study arm (healthy meals, produce box, and matched controls [standard of care]), nutrition counseling and education, and time (pre-intervention, 16 weeks, six months and 12 months). The primary dependent variable (outcome) is absolute weight change at 16 weeks between groups. The secondary dependent variables (outcomes) are as follows: i) absolute weight change at six months and 12 months from baseline; ii) change in hbA1c, dosage, and number of diabetes medications; iii) change in dosage and number of blood pressure medications, and; iv) change in numeric rating from baseline to 16 weeks for food insecurity, diet quality, depression as well as self-efficacy for health eating, healthy weight, and chronic disease management. A final outcome of interest is satisfaction rating for the interventions provided in the study, measured using a non-validated questionnaire developed for this study.

Descriptive statistics will be calculated for demographic data and for each dependent variable by group, nutrition counseling and education, and time. Separate repeated-measures ANOVAs will be conducted for each dependent variable with the between subjects factor group (healthy meals, produce box, and matched controls) and the within subjects factor time (pre-intervention and 16 weeks). *Post hoc* testing will be conducted as appropriate. Effect sizes will be calculated between pre-intervention and 16 weeks to capture the changes from pre- to post-intervention. Missing data will be analyzed using an intention-to-treat strategy. Statistical analysis will be performed by a biostatistician using SAS statistical software. The biostatistician will be blinded to the intervention for each group to eliminate potential bias during data analysis.

## Discussion

While scholarship on direct provision of healthy foods to patients continues to grow (Downer *et al.*, 2020; Veldheer *et al.*, 2020), past research in this area has been limited by singular rather than comparative testing of the effects of different interventions on health outcomes. In turn, our understanding of how different interventions, for example, medically tailored meals or prescription produce programs, stack up against one another is lacking. Furthermore, previous studies have not always incorporated nutrition education as part of healthy food interventions, a component critical to helping participants gain or increase knowledge on nutritious foods, portion sizes, and how to tailor their diet to underlying medical conditions or contextual factors such as financial constraints or the seasonality of produce. This food as medicine study was developed to address these gaps in the literature. To our knowledge, this study is the first quasi-experimental study to test two kinds of healthy food interventions – ready-made healthy meals and produce – compared to standard of care, using a parallel-arm design with participants drawn from the same population.

Adopting a healthy diet is a key lifestyle modification in the management of obesity and adiposity-linked chronic diseases. A modest weight loss of 5% of body weight has been shown to improve chronic conditions, with higher weight loss correlated with greater health outcomes (Jensen *et al.*, 2014; Ryan and Yockey, 2017). While various dietary strategies to promote weight loss exist, in general, reduced-calorie diets that are unrestrictive of macronutrient type (Sacks *et al.*, 2009) and that encourage increased dietary fiber consumption (Slavin, 2005; Anderson *et al.*, 2009) appear best suited to aid weight loss. In the landmark POUNDS Lost study, dietary fiber intake promoted weight loss, irrespective of macronutrient intake in overweight and obese adults consuming a calorie-restricted diet (Sacks *et al.*, 2009; Miketinas *et al.*, 2019). Thus, by providing food items comprised of adequate daily dietary fiber to our study participants, it is plausible that they can achieve modest weight loss within a period of 16 weeks. In this food as medicine study, the comparison between structured daily healthy meals and produce box versus standard of care is intended to assess the effect size of weight loss across study arms. Past research has demonstrated that pre-packaged portion-controlled meals versus self-selected diets resulted in weight loss among overweight or obese adults at eight weeks (Hannum *et al.*, 2004) and at 12 weeks (Rock *et al.*, 2016).

Our study’s exploratory outcomes will elucidate the effect of each intervention on diabetes and blood pressure control at 16 weeks, six months, and 12 months from baseline. Prior research on cardiometabolic changes associated with weight loss has typically utilized a single long-term time point, for example, 12 months or 24 months (Wing *et al.*, 2011; Dow *et al.*, 2013; Ahmad Zamri *et al.*, 2020; Iwamoto *et al.*, 2021); therefore, there is need to examine the cardiometabolic endpoints of weight loss in the short term and medium term. Finally, findings on food insecurity, diet quality, depression, self-efficacy for health eating, healthy weight, and chronic disease management, as well as participants’ adherence to, and satisfaction with, study interventions, will add to the mounting evidence on the impact of direct provision of healthy foods (Palar *et al.*, 2017; Berkowitz *et al.*, 2020; Wu *et al.*, 2022; Jd Steer *et al.*, 2023).

## Strengths and limitations

The main strength of this study is the quasi-experimental design comparing two parallel groups against matched controls, with biomarkers assessed soon after the completion of the interventions. A second strength of the study is the assessment of the effect of the tested interventions on other domains such as food insecurity, diet quality, mood, and self-efficacy for healthy eating and chronic disease management using validated instruments. In a review of health care-linked programs to increase access to fruits and vegetables, Veldheer *et al.* (2020) found that such programs lacked rigorous design and did not always collect biomarkers or utilize validated instruments. The aforementioned strengths of our study, thus, address key shortcomings of previous interventions. A third strength of the study is its attention to affordability and access issues, which are known contributors to poor nutrition (Larson *et al.*, 2009; Darmon and Drewnowski, 2015; CDC, 2020), and the consideration of which constitutes patient-centered strategies to promote healthy eating (Saver *et al.*, 2015). Accordingly, interventions tested in this study will be offered at no cost to participants, with food items shipped directly to the participants’ residence for approximately four months.

One limitation of this study is the relatively small sample size of 70 participants who will receive interventions. Consequently, participant attrition from the study will threaten the study power. A second limitation of the study is the lack of a parallel control group. Study controls will consist of 70 matched participants identified via the electronic health record from the same patient population as the intervention arm participants. Due to funding and ethical reasons, this study is unable to utilize a true control group for comparison with the intervention groups. Our study population faces many social challenges, including food insecurity; thus, it is important to ensure that would-be controls receive compensation comparable to the healthy food items received by intervention arm participants. The small amount of the study grant limits the ability to compensate or offer equivalent healthy food items to controls after the intervention period. A third limitation of this study is the relatively short timeline (16 weeks) in which the primary outcome will be assessed. Because this study will offer interventions for only 15 weeks, and to guard against the high attrition common in studies with a lengthy period of follow-up, assessing the primary outcome soon after completion of the interventions is deemed appropriate. With the expected weight loss of one to two pounds a week over the 15-week intervention period, the timeline for the primary outcome is still clinically meaningful. Additionally, biomarkers of interest will be collected via review of the electronic medical record at six months and 12 months from baseline. While these data will allow for the assessment of study outcomes longitudinally, a significant amount of missing data will hinder such a pursuit. A final limitation of the study is the lack of blinding to treatment arm assignment among assessors collecting self-reported data. To mitigate this limitation, during the baseline visit, self-reported data will be collected prior to random allocation to intervention arms. In addition, the biostatistician analyzing the data will be blinded to the treatment arms. Despite the aforementioned limitations, we anticipate that the results of this study will add to the growing body of scholarship on the impact of direct healthy food interventions on chronic diseases.

## Conclusion

Direct provision of healthy foods for the prevention and management of chronic diseases is an emerging area of study that warrants further investigation. Specifically, the health outcomes associated with different healthy food interventions is of high interest as such insight can guide providers in tailoring their recommendations for healthy food options to patients. This food as medicine project was designed with this objective in mind, and the study results are expected to offer comparative health outcomes between two common direct interventions – healthy meals and produce.

## References

[ref1] Ahmad Zamri L , Appannah G , Zahari Sham SY , Mansor F , Ambak R , Mohd Nor NS and Aris T 2020. Weight change and its association with cardiometabolic risk markers in overweight and obese women. Journal of Obesity 2020, 1–10. 10.1155/2020/3198326 PMC7211250 32399286

[ref2] Ahnen RT , Jonnalagadda SS and Slavin JL 2019. Role of plant protein in nutrition, wellness, and health. Nutrition Reviews 77, 735–747. 31322670 10.1093/nutrit/nuz028

[ref3] Anderson JW , Baird P , Davis RH , Ferreri S , Knudtson M , Koraym A , Waters V and Williams CL 2009. Health benefits of dietary fiber. Nutrition Reviews 67, 188–205. 19335713 10.1111/j.1753-4887.2009.00189.x

[ref4] Bandura A 2004. Health promotion by social cognitive means. Health Education & Behavior 31, 143–164. 15090118 10.1177/1090198104263660

[ref5] Berkowitz SA , Delahanty LM , Terranova J , Steiner B , Ruazol MP , Singh R , Shahid NN and Wexler DJ 2019. Medically tailored meal delivery for diabetes patients with food insecurity: a randomized cross-over trial. Journal of General Internal Medicine 34, 396–404. 30421335 10.1007/s11606-018-4716-z PMC6420590

[ref6] Berkowitz SA , O’Neill J , Sayer E , Shahid NN , Petrie M , Schouboe S , Saraceno M and Bellin R 2019. Health center-based community-supported agriculture: an RCT. American Journal of Preventive Medicine 57, S55–S64. 31522922 10.1016/j.amepre.2019.07.015 PMC6874748

[ref7] Berkowitz SA , Shahid NN , Terranova J , Steiner B , Ruazol MP , Singh R , Delahanty LM and Wexler DJ 2020. “I was able to eat what I am supposed to eat”-- patient reflections on a medically-tailored meal intervention: a qualitative analysis. BMC Endocrine Disorders 20, 10. 31959176 10.1186/s12902-020-0491-z PMC6971854

[ref8] Berkowitz SA , Terranova J , Hill C , Ajayi T , Linsky T , Tishler LW and DeWalt DA 2018. Meal delivery programs reduce the use of costly health care in dually eligible medicare and medicaid beneficiaries. Health Affairs 37, 535–542. 29608345 10.1377/hlthaff.2017.0999 PMC6324546

[ref9] Berkowitz, SA , Terranova J , Randall L , Cranston K , Waters DB and Hsu J 2019. Association between receipt of a medically tailored meal program and health care use. JAMA Internal Medicine 179, 786. 31009050 10.1001/jamainternmed.2019.0198 PMC6547148

[ref10] Borgonovi TF , Virgolin LB , Janzantti NS , Casarotti SN and Penna ALB 2022. Fruit bioactive compounds: effect on lactic acid bacteria and on intestinal microbiota. Food Research International 161, 111809. 36192952 10.1016/j.foodres.2022.111809

[ref11] Cavanagh M , Jurkowski J , Bozlak C , Hastings J and Klein A 2017. Veggie Rx: an outcome evaluation of a healthy food incentive programme. Public Health Nutrition 20, 2636–2641. 27539192 10.1017/S1368980016002081 PMC5743436

[ref12] CDC 2020. Access to healthy foods. Centers for Disease Control and Prevention [Online]. [Accessed 13 March 2023]. Available from: https://www.cdc.gov/nutrition/healthy-food-environments/improving-access-to-healthier-food.html.

[ref13] Cena H and Calder PC 2020. Defining a healthy diet: evidence for the role of contemporary dietary patterns in health and disease. Nutrients 12, 334. 32012681 10.3390/nu12020334 PMC7071223

[ref14] Chan A-W , Tetzlaff JM , Altman DG , Laupacis A , Gøtzsche PC , Krleža-Jerić K , Hróbjartsson A , Mann H , Dickersin K , Berlin JA , Doré CJ , Parulekar WR , Summerskill WSM , Groves T , Schulz KF , Sox HC , Rockhold FW , Rennie D and Moher D 2013. SPIRIT 2013 statement: defining standard protocol items for clinical trials. Annals of Internal Medicine 158, 200. 23295957 10.7326/0003-4819-158-3-201302050-00583 PMC5114123

[ref15] Cleveland Clinic Akron General 2019. *Community health needs assessment* [Online]. [Accessed 13 March 2023]. Available from: https://my.clevelandclinic.org/-/scassets/files/org/about/community-reports/chna/2019/2019-akron-general-chna.pdf?la=en.

[ref16] Darmon N and Drewnowski A 2015. Contribution of food prices and diet cost to socioeconomic disparities in diet quality and health: a systematic review and analysis. Nutrition Reviews 73, 643–660. 26307238 10.1093/nutrit/nuv027 PMC4586446

[ref17] Ding L , Zhang L , Wen M , Che H , Du L , Wang J , Xue C , Xu J and Wang Y 2017. Eicosapentaenoic acid-enriched phospholipids improve atherosclerosis by mediating cholesterol metabolism. Journal of Functional Foods 32, 90–97.

[ref18] Dow CA , Thomson CA , Flatt SW , Sherwood NE , Pakiz B and Rock CL 2013. Predictors of improvement in cardiometabolic risk factors with weight loss in women. Journal of the American Heart Association 2, e000152. 24351700 10.1161/JAHA.113.000152 PMC3886783

[ref19] Downer S , Berkowitz SA , Harlan TS , Olstad DL and Mozaffarian D 2020. Food is medicine: actions to integrate food and nutrition into healthcare. BMJ 369, m2482. 32601089 10.1136/bmj.m2482 PMC7322667

[ref20] GBD 2017 Diet Collaborators 2019. Health effects of dietary risks in 195 countries, 1990–2017: a systematic analysis for the Global Burden of Disease Study 2017. The Lancet 393, 1958–1972. 10.1016/S0140-6736(19)30041-8 PMC6899507 30954305

[ref21] Hager ER , Quigg AM , Black MM , Coleman SM , Heeren T , Rose-Jacobs R , Cook JT , de Cuba SAE , Casey PH , Chilton M , Cutts DB , Meyers AF and Frank DA 2010. Development and validity of a 2-item screen to identify families at risk for food insecurity. Pediatrics 126, e26–e32. 20595453 10.1542/peds.2009-3146

[ref22] Hannum SM , Carson L , Evans EM , Canene KA , Petr EL , Bui L and Erdman JW 2004. Use of portion-controlled entrees enhances weight loss in women. Obesity Research 12, 538–546. 15044672 10.1038/oby.2004.61

[ref23] Harris PA , Taylor R , Minor BL , Elliott V , Fernandez M , O’Neal L , McLeod L , Delacqua G , Delacqua F , Kirby J and Duda SN 2019. The REDCap consortium: Building an international community of software platform partners. Journal of Biomedical Informatics 95, 103208. 31078660 10.1016/j.jbi.2019.103208 PMC7254481

[ref24] Harris PA , Taylor R , Thielke R , Payne J , Gonzalez N and Conde JG 2009. Research electronic data capture (REDCap)—a metadata-driven methodology and workflow process for providing translational research informatics support. Journal of Biomedical Informatics 42, 377–381. 18929686 10.1016/j.jbi.2008.08.010 PMC2700030

[ref25] Iwamoto SJ , Abushamat LA , Zaman A , Millard AJ and Cornier M-A 2021. Obesity management in cardiometabolic disease: state of the art. Current Atherosclerosis Reports 23, 59. 34345933 10.1007/s11883-021-00953-0 PMC8358925

[ref26] Jd Steer K , Olstad DL , Jt Campbell D , Beall R , Anstruther SM , Caron-Roy S and Spackman E 2023. The impact of providing material benefits to improve access to food on clinical parameters, dietary intake, and household food insecurity in people with diabetes: a systematic review with narrative synthesis. Advances in Nutrition 14, 1067–1084. 37245685 10.1016/j.advnut.2023.05.012 PMC10509434

[ref27] Jennings A , MacGregor A , Welch A , Chowienczyk P , Spector T and Cassidy A 2015. Amino acid intakes are inversely associated with arterial stiffness and central blood pressure in women. The Journal of Nutrition 145, 2130–2138. 26203100 10.3945/jn.115.214700 PMC4548168

[ref28] Jensen MD , Ryan DH , Apovian CM , Ard JD , Comuzzie AG , Donato KA , Hu FB , Hubbard VS , Jakicic JM , Kushner RF , Loria CM , Millen BE , Nonas CA , Pi-Sunyer FX , Stevens J , Stevens VJ , Wadden TA , Wolfe BM and Yanovski SZ 2014. 2013 AHA/ACC/TOS guideline for the management of overweight and obesity in adults: a report of the American College of Cardiology/American Heart Association Task Force on Practice Guidelines and The Obesity Society. Circulation 129, S102–S138. 24222017 10.1161/01.cir.0000437739.71477.ee PMC5819889

[ref29] Joint WHO/FAO Expert Consultation (ed.). 2003. Diet, nutrition, and the prevention of chronic diseases: report of a WHO-FAO Expert Consultation; [Joint WHO-FAO Expert Consultation on Diet, Nutrition, and the Prevention of Chronic Diseases, 2002, Geneva, Switzerland]. Geneva: World Health Organization.

[ref30] Kroenke K , Strine TW , Spitzer RL , Williams JBW , Berry JT and Mokdad AH 2009. The PHQ-8 as a measure of current depression in the general population. Journal of Affective Disorders 114, 163–173. 18752852 10.1016/j.jad.2008.06.026

[ref31] Larson NI , Story MT and Nelson MC 2009. Neighborhood environments. American Journal of Preventive Medicine 36, 74-81.e10. 10.1016/j.amepre.2008.09.025 18977112

[ref32] Liu RH 2013. Dietary bioactive compounds and their health implications: dietary bioactive compounds and health. Journal of Food Science 78, A18–A25. 23789932 10.1111/1750-3841.12101

[ref33] Lonnie M , Hooker E , Brunstrom J , Corfe B , Green M , Watson A , Williams E , Stevenson E , Penson S and Johnstone A 2018. Protein for life: review of optimal protein intake, sustainable dietary sources and the effect on appetite in ageing adults. Nutrients 10, 360. 29547523 10.3390/nu10030360 PMC5872778

[ref34] Luo C , Zhang Y , Ding Y , Shan Z , Chen S , Yu M , Hu FB and Liu L 2014. Nut consumption and risk of type 2 diabetes, cardiovascular disease, and all-cause mortality: a systematic review and meta-analysis. The American Journal of Clinical Nutrition 100, 256–269. 24847854 10.3945/ajcn.113.076109

[ref35] Malik VS , Li Y , Tobias DK , Pan A and Hu FB 2016. Dietary protein intake and risk of type 2 diabetes in US men and women. American Journal of Epidemiology 183, 715–728. 27022032 10.1093/aje/kwv268 PMC4832052

[ref36] Manuelli M , Della Guardia L and Cena H 2017. Enriching diet with n-3 PUFAs to help prevent cardiovascular diseases in healthy adults: results from clinical trials. International Journal of Molecular Sciences 18, 1552. 28718800 10.3390/ijms18071552 PMC5536040

[ref37] Micha R , Shulkin ML , Peñalvo JL , Khatibzadeh S , Singh GM , Rao M , Fahimi S , Powles J and Mozaffarian D 2017. Etiologic effects and optimal intakes of foods and nutrients for risk of cardiovascular diseases and diabetes: Systematic reviews and meta-analyses from the Nutrition and Chronic Diseases Expert Group (NutriCoDE) S. Kiechl, ed. PLOS ONE 12, e0175149. 28448503 10.1371/journal.pone.0175149 PMC5407851

[ref38] Miketinas DC , Bray GA , Beyl RA , Ryan DH , Sacks FM and Champagne CM 2019. Fiber intake predicts weight loss and dietary adherence in adults consuming calorie-restricted diets: the POUNDS lost (preventing overweight using novel dietary strategies) study. The Journal of Nutrition 149, 1742–1748. 31174214 10.1093/jn/nxz117 PMC6768815

[ref39] Mozaffarian D , Mande J and Micha R 2019. Food is medicine—the promise and challenges of integrating food and nutrition into health care. JAMA Internal Medicine 179, 793. 31009044 10.1001/jamainternmed.2019.0184

[ref40] Nair B 2019. Clinical trial designs. Indian Dermatology Online Journal 10, 193. 30984604 10.4103/idoj.IDOJ_475_18 PMC6434767

[ref41] Palar K , Napoles T , Hufstedler LL , Seligman H , Hecht FM , Madsen K , Ryle M , Pitchford S , Frongillo EA and Weiser SD 2017. Comprehensive and medically appropriate food support is associated with improved HIV and diabetes health. Journal of Urban Health 94, 87–99. 28097614 10.1007/s11524-016-0129-7 PMC5359179

[ref42] Ritter PL and Lorig K 2014. The English and Spanish Self-Efficacy to Manage Chronic Disease Scale measures were validated using multiple studies. Journal of Clinical Epidemiology 67, 1265–1273. 25091546 10.1016/j.jclinepi.2014.06.009

[ref43] Rock CL , Flatt SW , Pakiz B , Barkai H-S , Heath DD and Krumhar KC 2016. Randomized clinical trial of portion-controlled prepackaged foods to promote weight loss: Portion-controlled prepackaged foods and weight loss. Obesity 24, 1230–1237. 27225596 10.1002/oby.21481 PMC5312668

[ref44] Ryan DH and Yockey SR 2017. Weight loss and improvement in comorbidity: differences at 5%, 10%, 15%, and over. Current Obesity Reports 6, 187–194. 28455679 10.1007/s13679-017-0262-y PMC5497590

[ref45] Sacks FM , Bray GA , Carey VJ , Smith SR , Ryan DH , Anton SD , McManus K , Champagne CM , Bishop LM , Laranjo N , Leboff MS , Rood JC , De Jonge L , Greenway FL , Loria CM , Obarzanek E and Williamson DA 2009. Comparison of weight-loss diets with different compositions of fat, protein, and carbohydrates. New England Journal of Medicine 360, 859–873. 19246357 10.1056/NEJMoa0804748 PMC2763382

[ref46] Saver BG , Martin SA , Adler RN , Candib LM , Deligiannidis KE , Golding J , Mullin DJ , Roberts M and Topolski S 2015. Care that matters: quality measurement and health care. PLOS Medicine 12, e1001902. 26574742 10.1371/journal.pmed.1001902 PMC4648519

[ref47] Shang X , Scott D , Hodge A , English DR , Giles GG , Ebeling PR and Sanders KM 2017. Dietary protein from different food sources, incident metabolic syndrome and changes in its components: An 11-year longitudinal study in healthy community-dwelling adults. Clinical Nutrition 36, 1540–1548. 27746001 10.1016/j.clnu.2016.09.024

[ref48] Slavin JL 2005. Dietary fiber and body weight. Nutrition 21, 411–418. 15797686 10.1016/j.nut.2004.08.018

[ref49] Stella AB , Cappellari GG , Barazzoni R and Zanetti M 2018. Update on the impact of omega 3 fatty acids on inflammation, insulin resistance and sarcopenia: a review. International Journal of Molecular Sciences 19, 218. 29324650 10.3390/ijms19010218 PMC5796167

[ref50] Tufts University Friedman School of Nutrition Science and Policy 2023. Public impact initiative: food is medicine | Friedman School of Nutrition Science and Policy. [Accessed 13 March 2023]. Available from: https://nutrition.tufts.edu/about/public-impact-initiative-friedman-school/food-is-medicine.

[ref51] Vasmehjani AA , Darabi Z , Nadjarzadeh A , Mirzaei M and Hosseinzadeh M 2021. The relation between dietary phytochemical index and metabolic syndrome and its components in a large sample of Iranian adults: a population-based study. BMC Public Health 21, 1587. 34429094 10.1186/s12889-021-11590-2 PMC8383421

[ref52] Veldheer S , Scartozzi C , Knehans A , Oser T , Sood N , George DR , Smith A , Cohen A and Winkels RM 2020. A systematic scoping review of how healthcare organizations are facilitating access to fruits and vegetables in their patient populations. The Journal of Nutrition 150, 2859–2873. 32856074 10.1093/jn/nxaa209

[ref53] Wilson-Barlow L , Hollins TR and Clopton JR 2014. Construction and validation of the healthy eating and weight self-efficacy (HEWSE) scale. Eating Behaviors 15, 490–492. 25064304 10.1016/j.eatbeh.2014.06.004

[ref54] Wing RR , Lang W , Wadden TA , Safford M , Knowler WC , Bertoni AG , Hill JO , Brancati FL , Peters A , Wagenknecht L and the Look AHEAD Research Group 2011. Benefits of modest weight loss in improving cardiovascular risk factors in overweight and obese individuals with type 2 diabetes. Diabetes Care 34, 1481–1486. 21593294 10.2337/dc10-2415 PMC3120182

[ref55] World Health Organization 2013. Global action plan for the prevention and control of noncommunicable diseases 2013-2020 [Online]. Geneva: World Health Organization. [Accessed 26 February 2023]. Available from: https://apps.who.int/iris/handle/10665/94384.

[ref56] World Health Organization 2018. Healthy diet. [Accessed 26 February 2023]. Available from: https://www.who.int/publications/m/item/healthy-diet-factsheet394.

[ref57] Wu JH , Trieu K , Coyle D , Huang L , Wijesuriya N , Nallaiah K , Lung T , Di Tanna GL , Zheng M , Mozaffarian D , MacMillan F , Simmons D , Wu T , Twigg S , Gauld A , Constantino M , McGill M , Wong J and Neal B 2022. Testing the feasibility and dietary impact of a “Produce Prescription” Program for adults with undermanaged type 2 diabetes and food insecurity in Australia. The Journal of Nutrition 152, 2409–2418. 36774107 10.1093/jn/nxac152

[ref58] Zhang T-T , Xu J , Wang Y-M and Xue C-H 2019. Health benefits of dietary marine DHA/EPA-enriched glycerophospholipids. Progress in Lipid Research 75, 100997. 31442526 10.1016/j.plipres.2019.100997

